# Facilitating active participation in anticoagulant decisions in advanced kidney disease: co-production of a question prompt list

**DOI:** 10.1186/s12882-025-03966-y

**Published:** 2025-01-28

**Authors:** Kathrine Parker, Abigail Needham, Jecko Thachil, Sandip Mitra, Penny Lewis

**Affiliations:** 1 Division of Pharmacy and Optometry, School of Health Sciences, The University of Manchester, University of Manchester, Manchester, M13 9PT UK; 2 Manchester Institute of Nephrology and Transplantation, Manchester University NHS Foundation Trust, Oxford Road, Manchester, M13 9WL UK; 3 National Institute for Healthcare Research Devices for Dignity MedTech Co-operative, Sheffield Teaching Hospitals NHS Foundation Trust, Sheffield, S10 2JF UK; 4 Department of Haematology, Manchester University NHS Foundation Trust, Oxford Road, Manchester, M13 9WL UK; 5 Division of Cardiovascular Sciences, School of Medical Sciences, The University of Manchester, Manchester, M13 9NT UK; 6 The University of Manchester, Division of Pharmacy and Optometry, School of Health Sciences, Manchester, M13 9PT UK

**Keywords:** Anticoagulation, Chronic Kidney Diease, Co-production, Shared-decision making, Patient involvement

## Abstract

**Background:**

People with chronic kidney disease are at increased risk of thrombotic and bleeding episodes making anticoagulant treatment decisions challenging. Currently, there are no support tools for people with chronic kidney disease regarding anticoagulant therapy decisions. This work aimed to co-produce materials to support shared-decision making when considering anticoagulant use in advanced chronic kidney disease.

**Methods:**

Focus groups were undertaken to explore the views of people with kidney disease towards anticoagulant prescribing. Data was thematically analysed based on Makoul and Clayman’s model of shared-decision making. Co-production methods were used to develop a question prompt list based on themes from the focus groups in conjunction with people with kidney disease over three meetings.

**Results:**

A question prompt list, to be used by patients when initiated on anticoagulant therapy, was co-produced. These questions were based upon participants’ experiences of the various stages of shared-decision making within the context of anticoagulant use in advanced chronic kidney disease. Of particular importance to participants was the individualised discussion around treatment risks and follow up arrangements.

**Conclusion:**

Shared-decision making is important when initiating medication to ensure the best outcomes for patients, yet it can be difficult to engage in shared-decision making without prompts or guidance. This co-produced question prompt list could be included as part of national guideline to support shared-decision making for anticoagulant initiation in patients with advanced chronic kidney disease.

**Supplementary Information:**

The online version contains supplementary material available at 10.1186/s12882-025-03966-y.

## Background

Approximately 6% of adults in the United Kingdom live with Chronic Kidney Disease (CKD) [1]. People with CKD have an increased risk of atrial fibrillation (AF), which can lead to ischaemic stroke [2–4]. Stroke is the second leading cause of death globally and approximately 50% of survivors remain chronically disabled [5]. People with AF usually warrant prophylactic anticoagulation, however, there are gaps in the evidence for people with advanced CKD, eGFR < 30ml/min/1.73m^2^. For example, it is unclear whether anticoagulants reduce stroke risk in those on dialysis [6].

People with CKD are at greater risk of thrombotic [7, 8] and bleeding episodes [9–11] than those with normal kidney function. There exists a delicate balance between bleeding risk and thrombosis prevention, making anticoagulant treatment decisions challenging [12]. Previous research has reported that patients commencing anticoagulant treatment for AF are more concerned about stroke than the risk of bleeding [13, 14]. However, these findings may not extrapolate to a CKD population who are at an elevated risk of bleeding.

Patients should be encouraged to be involved in treatment decisions in a collaborative process of shared-decision making (SDM) with healthcare professionals [15, 16]. SDM facilitates patient understanding and medication-taking [17], optimising medication use for best possible outcomes [18]. This process requires clear, evidence-based information about treatment options and outcomes, however, as evidence regarding anticoagulation therapy in CKD is limited, supporting patients to make informed decisions is difficult. Question Prompt Lists (QPLs) can reduce disconnect between the patient and prescriber by enabling provision of information that address patients’ main priorities and therefore reduce patient anxieties [19]. However, at present there is no support available for patients with CKDwhen initiating anticoagulants.

The aim of this work was to co-produce materials that can support SDM for anticoagulant use in CKD to be used by clinicians and patients.

## Methods

### Study design

This study followed the principles of co-production, with patients and healthcare professionals working as partners in developing patient information [20] and with patient and public involvement (PPI) throughout all stages of the study [21]. Focus groups were undertaken to allow people with lived experience of CKD taking anticoagulants to discuss their experiences with others, encouraging openness and allowing participants to build on each other’s ideas [22].

### Focus group participants and recruitment

Participants were eligible to take part in focus groups if they were over 18 years old, were currently or previously taking an oral anticoagulant (including edoxaban, apixaban, rivaroxaban dabigatran or warfarin) whilst under the care of a nephrologist with CKD stage 4 or 5 [23]. Adverts were disseminated via thrombosis and kidney patient networks.

### Focus group data collection

A semi-structured topic guide, found in supplementary appendix one, was developed in conjunction with two people with kidney disease who had experience of taking anticoagulants. They were able to support with topics to include and the questions (wording and order) to ensure patients’ views of information provision and personal experiences regarding anticoagulation treatment were explored in depth. The questions fell into the categories described by Leung and Savithiri [22] including opening questions, introductory questions and the key questions of interest. Consent was taken by KP prior to the focus groups. Two focus groups, with an optimal number of between 4–5 participants [24], were conducted via Microsoft Teams and each was led by KP and a member of National Institute Healthcare Research (NIHR) dignity for devices renal theme, AN.

### Focus group data analysis

Focus groups were audio recorded and transcribed verbatim. Transcripts were checked for accuracy by KP before being de-identified and imported into NVivo, a qualitative data software package, which supported data management during analysis. A hybrid approach to thematic analysis was undertaken, with data deductively analysed based on a model of key elements of SDM derived by Makoul and Clayman [25] and inductively, to ensure new themes, not captured within the model, were incorporated. KP and PL read the transcripts independently and discussed emerging themes. Analysis was an iterative process, with refinement of coding and grouping of themes until agreement was reached between KP and PL.

The model of SDM by Makoul and Clayman was developed based on a review of existing literature on SDM in medical encounters and was selected as a framework for analysis as it incorporates the essential elements of SDM that clinicians should follow during a consultation [25]. The coding framework can be found in supplementary appendix two.

### Development of question prompt list

**Fig. 1:**
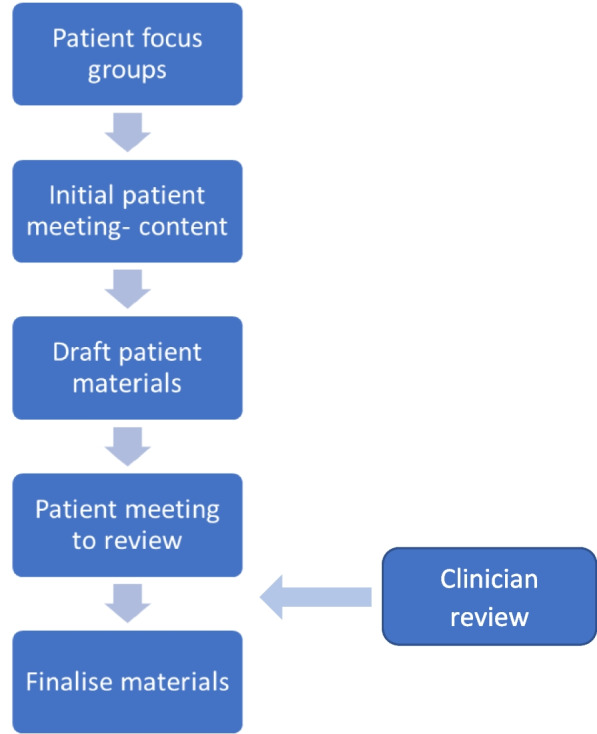
Steps involved in co-production of Question Prompt List

Development of the QPL was an iterative process, undertaken in conjunction with kidney patients and the lead researcher, KP, a kidney pharmacist (Fig. 1). Six participants from the focus groups supported the development and revisions of the final co-produced QPL, which involved three meetings with e-mail correspondence between each meeting. The themes from the focus groups were used to develop the questions in conjunction with the participants.

The first meeting included five people with kidney disease and discussions focussed upon views of existing published patient information and the desired content and format of future patient information. Following the initial meeting, a draft document was produced by KP which included basic anticoagulant information and a list of questions which was derived from the main focus group themes. The draft was shared with patients prior to a second meeting with five patients, during which the draft was revised based on patient feedback. This version of the document was shared with a nephrologist, SM, and haematologist, JT, for their feedback. So that patients were comfortable expressing their views, feedback from healthcare professionals took place separately towards the end of the process – this ensured patients were not influenced or hindered by their presence [22]. A third meeting took place, during which three participants reviewed and agreed that this was the final document.

Patients were reimbursed for PPI activities at INVOLVE rates [26]. Ethical approval for this study was granted by North-West - Greater Manchester Central Research Ethics Committee. REC reference: 21/NW/0180.

## Results

Nine participants took part in two focus groups, lasting between 90–120 minutes, participant demographics are detailed in Table 1. All participants with a kidney transplant had been taking an anticoagulant whilst on dialysis or had kidney function that met the eligibility criteria. Four patients who consented were unable to take part- one had died and three were hospitalised. Despite this data saturation was achieved [27].

**Table 1:** Participant demographics

Demographics	Number of patients N
Sex (Male/Female)	(3/6)
Median duration anticoagulation (IQR), years	11.5 (4–22.5)
**Current kidney function**	
Dialysis	5
CKD stage 4	1
Kidney transplant	3
**Indication**	
Pulmonary Embolism	1
Deep Vein thrombosis	2
Recurrent Deep Vein Thrombosis	1
Metallic valve	1
Atrial Fibrillation	3
Chronic Thromboembolic Pulmonary Hypertension	1
**Location**	
North East	2
North West	2
East Midlands	1
Northern Ireland	1
London	2
South East England	1
**Current anticoagulation**	
Warfarin	8
Direct Oral Anticoagulant	1

*IQR* Interquartile Range 25th −75th Quartiles

All themes from Makoul and Clayman’s model of SDM were identified, with no additional themes. Each theme is described in the order presented in the SDM model, along with illustrative quotes. The co-produced questions based on each theme, for inclusion in the document, are found in Table 2.

### Explanation of the problem

The majority of participants understood why they were prescribed an anticoagulant with the reasons being described to them by the prescriber. Patients with AF were provided with an explanation of the risk of stroke to support their understanding of why treatment is lifelong. However, three participants said there had been no discussion of treatment duration with their clinician and assumed treatment would be for life:

*“You know, I, I assume it’s for life, but I, I don’t know, no one’s ever had that discussion really”. Patient 1, Female*

Understanding the duration of treatment was important to patients and this was described by one participant, who felt it would have influenced their engagement with discussions regarding their ongoing treatment:

*“…to find out if it’s [anticoagulation] long term or not. If told it is going to be long-term, then, eh, then immediately push for, okay, this is going to have a large effect on my life. I want to be part of that journey.” Patient 2, Male*

### Presentation of treatment options and monitoring

Treatment options were not always presented to participants. However, participants wanted to know what treatments were available, whether they were eligible for them and the reasons for this.

Most participants felt unprepared for the burden of warfarin monitoring. One participant described the difficulties she experienced with International Normalised Ratio (INR) monitoring and the negative impact this had on her daily life:

*“…I didn’t drive. And of course, you have to get it done first thing in the morning, and I had to drop one child off at school, and bring the other one with me, and walk through every weather that existed, for months, and months, and months, every single week to go and get it done…And that’s probably, out of like all of the things that have happened to me, medically, that’s one of the hardest.” Patient 3, female*

Differing local arrangements for INR monitoring were described and included monitoring via the dialysis unit, anticoagulant clinic, the General Practitioners or at home. For the majority of participants, monitoring options were not discussed at treatment initiation or at follow up. However, discussion of monitoring options at therapy initiation may have reduced the burden of treatment on participants’ lives and empowered patients by giving them a choice in where monitoring took place. This positive impact was highlighted by one participant who described his emotions when he found out he no longer needed to travel to hospital:

*"Finding out I could actually do it at my doctor’s was…I, I nearly cried because I didn’t have to go to hospital anymore and travel..." Patient 4, Male*

### Risks and benefits

Participants were certain anticoagulation was indicated when it was initiated and understood the benefits of treatment (supplementary appendix two). Bleeding was the most common treatment risk described by participants. However, for several participants, the first time they were aware of bleeding as a possible implication of anticoagulant treatment was when they experienced it. They did not recall receiving information about the risks of bleeding when the anticoagulant was initiated. One patient, prescribed warfarin, recalled his experiences of a bleeding episode:

*“ they [treatment risks] haven’t actually been formally explained to me. I think I’ve found them all, because, [of] incidences, like, sitting on that train and then suddenly looking down and watching, like, the… my shirt cuff [laughs] turn crimson!” Patient 2, Male*

Although the risk of bleeding may have been explained to some participants, the seriousness of potential bleeding was not clearly described. This participant describes learning about the risks of severe bleeding from the emergency team after a fall:

*“…they [healthcare team] talked about if you, if you had a head injury, about the possibilities of a bleed on the brain… And I think those sort of things, things that are quite critical, are probably very important to me.” Patient 1, female*

Three female participants highlighted how heavy menstrual bleeding, as a possible consequence of treatment, was not explained to them. The onus was on them to raise this issue rather than it being discussed. Despite highlighting their concerns to healthcare staff, two participants suffered major bleeding due to menses, resulting in multiple blood transfusions:

*“I was having a lot of problems with menstrual bleeding, and I told them that. But they just ignored it. And eventually, I became very ill because I lost so much blood, and [I] had a lot of blood transfusions, which compromised my position to have another transplant.” Patient 6, female*

### Factors affecting the risks of treatment

Participants wanted to receive more information on factors that could increase the risk of adverse effects, particularly with warfarin, where alcohol, certain foods, antibiotics and other interacting medicines can be hazardous. Patients on warfarin therapy also need to be aware that if they have a medical procedure (e.g. tooth extraction) that they may need an interruption of their anticoagulant therapy and bridging with an anticoagulant. All participants described how they were poorly informed of what was required in relation to anticoagulation when undergoing surgery and one participant experienced harm (DVT) as no bridging anticoagulant was given around the time of surgery. Participants also described scenarios where bridging had been poorly managed, and one participant had taken it upon themselves to devise their own bridging plan:

*“ … I had, ..surgery last year and it ended up with …I think the day before the operation, me going in, speaking to the anaesthetist, and we prepared our own plan…. And, and now that’s the way I treat it, I now go in and say, look, I take warfarin, I want a bridging plan before anything happens” Patient 2, Male*

Participants were not clear who was responsible for devising their bridging regime. This was particularly problematic when participants received care from different service providers.

### Professional’s recommendations

Although participants did not dispute the need for anticoagulant therapy, the way it was communicated could be described as paternalistic, with some participants describing feeling like they did not have a choice:

*“Doctors instantly go… this is what you need to do, this is where you need to go, this is what you’re going to say, this is how it’s going to go.” Patient 4, Male*

### Check understanding or defer follow up

At the time of anticoagulant initiation, many participants were hospitalised with severe illness and were coming to terms with their diagnosis. They found it difficult to comprehend information relating to anticoagulant therapy during hospitalisation and would have preferred to have discussions regarding therapy after being discharged from hospital, when they felt better able to engage with discussions and had the support of family or friends:

*“… a doctor tells you…’your life’s going to change for the rest of your life with, with the one tablet’. My issue is they keep talking instead of going, ‘right, do you understand this?’ No. ‘Go away, come back at another time with somebody else…that can support you and can digest the information I’m going to give you, because this is going to be something very important’…But doctors don’t do that” Patient 4, male*

Patients expressed a preference to takeaway information, particularly as some patients like to discuss the information with family or carers, prior to having a follow up appointment. One participant described how their wife was a major support to them when making decisions and interpreting information:

*“…what works for me, that…having a piece of paper with information, I would pass to my, my wife, and say, just read through this and understand it for me and tell me what I need to know and understand better.” Patient 4, male*

### Patient preferences

Patient preferences were implicit within previously described themes, however, preferences for the format of patient information were discussed. Written information in simple language was preferred by most, however, multiple formats of information, including videos and leaflets, were all deemed useful, appealing to different patient needs and facilitating reinforcement of important information.

### Arrange follow up

Participants described how they did not receive follow up information or have discussions about their anticoagulant therapy once it had been initiated, yet they would have valued the opportunity to revisit treatment and monitoring options:

*“…three and a half years down the line and nothing has ever been revisited since, in terms of, you know, are there alternatives? Is it, is it the right treatment?” Patient 1, female*

**Table 2:** Questions included in the final question prompt list relating to SDM themes

Theme	Questions
Explanation of the problem	Why am I taking an anticoagulant? How long will I need to take my anticoagulant for?
Presentation of treatment options and monitoring	What kinds of anticoagulants can be prescribed for me? What monitoring do I need to have, for example specific blood tests? Can I choose where this monitoring can be carried out?
Risks and benefits Factors affecting the risks	What are the main side effects? When do I need to seek medical attention? Does my diet or medicines affect my anticoagulant? What happens if I need a tooth removing or surgery?
Professional’s recommendations	None *There were no specific questions arising from this theme. Recommendations will be made in response to the previous questions to support patient decision-making.*
Check understanding or defer follow up	None *This requires clinicians to gauge whether a patient requires time to digest information and/or discuss with family/carers, as well as frank honesty from the patient highlighting to clinicians that they would like to discuss this further or at a later time*
Patient preferences	Who will follow up on my treatment? *Includes questions under the theme treatment and monitoring preferences. The final co-produced QPL also contains a list of resources for patients to access specific to their needs.*
Arrange follow up	Who will follow up on my treatment? Who can I contact if I need help or advice?

### Findings resulting in the development of the question prompt list

As described in the methods, the development of the QPL involved three meetings. In the first meeting, the patients made the decision that a QPL should be produced to guide discussions with clinicians when anticoagulation was initiated. A QPL would ensure information was individualised for each patient. During the second meeting, the patient group decided to include a personalised regime section and resource list as well as making some language amendments. There were no amendments made to the document by the nephrologist and haematologist. The final meeting resulted in agreement on the final version which can be found in supplementary appendix three. The enablers for design and implementation of a QPL were considered when developing this document [28]. Enablers including having a complex clinical scenario where the QPL would support the discussion, ensuring there were not too many questions and making sure the QPL would be accessible for patients and clinicians were all considered.

As well as generating a QPL for use by people with kidney disease and carers, this study illuminated a set of recommendations (Table 3) for clinicians when having discussions around anticoagulant initiation. These recommendations have implications for all health professionals involved in the care of people with kidney disease and are derived from the themes of the focus groups and based upon SDM recommendations [29].

**Table 3:** Recommendations for health professionals in relation to SDM for use of anticoagulation in chronic kidney disease

Ask patients whether they would like a family member/carer involved in discussions
Assess whether patient (+/-family or carer) need more time to consider the decision and whether follow up is required
Depending on the needs of the patient, identify if they want to be involved in the decision or would prefer it to be made by clinician
Address any questions the patients +/- carers or family may have
For female patients of child-bearing age, enquire whether they still have menses and if so, discuss potential effect of anticoagulant therapy on menses
For patients on warfarin, ensure they receive a National Patient Safety Agency (NPSA) yellow book and information relating to what can affect INR along with information adverse effects
For patients on warfarin, discuss potential locations of where they could undertake monitoring
Explain who will follow up patients’ treatment and ways to contact them

## Discussion

The National Institute for Clinical Excellence (NICE) suggest that the first step in SDM is providing patients with information to help them consider what matters to them and questions they may like to ask [29]. In practice, providing information in advance to patients can be difficult. We have successfully co-produced with patients, a list of pre-formulated questions that can support SDM around anticoagulant use in CKD.

Each conversation around anticoagulation needs to take into consideration the patient’s individual information requirements and preferences. This practice is embedded into primary care with clinician use of the mnemonic, ICE- Ideas, Concerns and Expectations [30]. However, incorporating patients’ perspectives requires time in the consultation for best patient outcomes to be achieved [30]. Within the QPL, key questions are readily available should the patient wish to ask them. The extent of patient involvement in SDM is, however, varied and influenced by multiple factors such as the complexity of the decision, cultural and language barriers. Some patients prefer a non-participatory role in treatment decisions [31–35] and this can lead to prescribers taking on sole responsibility for treatment decisions [36]. Conversely, younger patients and those with a greater level of education have been reported to have an increased desire to be involved in treatment decisions [31, 37].

Patients within the study were not involved in the decision to start an anticoagulant, which corresponds to the ‘paternalistic model’ of care where clinicians make decisions for the patient [36]. There may be a multitude of reasons why clinicians may not fully embrace a SDM approach. It is well known that time is a huge barrier to SDM and, given current resource limitations within clinic settings, this may have influenced the clinician’s approach to the consultation [38–40]. Patients with kidney disease can be medically challenging, with a recent study from the US showing that patients seen by nephrologists had the highest number of co-morbidities, number of prescribed medicines and rate of death [41], adding significant complexity to decision making. This may lead clinicians to believe that patients are unable to interpret information and make such challenging and complex decisions. Additionally health literacy, has been shown to be limited in a CKD population, with 32% of a large haemodialysis cohort reported to have limited health literacy [42]. Available data shows an association with adverse events and healthcare use in those with CKD and limited health literacy [43, 44].

NICE SDM guidance suggests that the risks and benefits of possible treatments should be discussed openly [29]. One risk that was felt to be poorly explained to female participants was heavy menstrual bleeding. It is acknowledged that women’s health is poorly discussed, and this is a focus of the Department of Health and Social Care women’s healthy strategy [45]. A recent study in the general population, revealed that 60% of women initiated on an anticoagulant develop abnormal menstrual bleeding. This was associated with a reduction in quality of life when experiencing heavy menstrual bleeds [46], highlighting the need to increase patients’ awareness of the problem [46].

Information overload is defined as a situation whereby the volume of information supplied in a given time frame exceeds an individual’s capacity to process that information [47]. In such situations, an individual may fail to pay attention to information, incorrectly process information, or avoid information [48, 49]. Participants in this study described information overload at the time of anticoagulant initiation, including forgetting the information after it’s been provided and feeling overwhelmed. NICE suggest that people should be offered support to engage in decision-making [39] and this may include arranging a separate follow up appointment, offering tailored information, and inviting family/friends if requested. This fits the model of some cancer associated thrombosis clinics where patients initiated on an anticoagulant are followed up within a week to discuss any questions or concerns and to reinforce key messages around anticoagulant use [50], an approach that could be adopted in the renal outpatient setting. Asking a patient whether they want a carer or family member involved in the consultation, to facilitate SDM [39] may be especially pertinent in patients with advanced kidney disease as they can experience difficulty concentrating or memory problems (‘brain fog’) [51, 52], making it more difficult to comprehend and interpret information.

### Strengths and limitations

The study had good patient representation from across the United Kingdom. People were taking anticoagulation for a variety of reasons and these factors make this work applicable to a wide range of individuals with advanced kidney disease. Our co-production approach means that the QPL was designed and tailored for what patients really need, leading to the first document to support people with kidney disease in their decisions around anticoagulation.

There were only a small number of participants who took part in the focus groups, with four consented participants being unable to attend due to illness. It is well known that recruitment of people with chronic disease can be difficult [53, 54]. Despite this, themes were strong throughout both discussions and data saturation was reached. A limitation is that the data collection method required participants to be digitally enabled which may have excluded those not able to access technology. Furthermore, our participant group lacked diversity, with no representation of ethnic minority groups. These limitations were in part due to COVID-19, limiting our approach and ability to reach out to underserved groups in their community setting. Future research should explore the adaptation and acceptability of our QPL for ethnic minority groups which is important due to the emerging evidence that these groups experience inequity and are at higher risk of patient safety events [55].

Although this work has co-produced a QPL to support SDM, and recommendations for clinicians, both would need to be tested in the clinical setting for their acceptability to ensure effective implementation.

## Conclusion

Shared-decision making is important to ensure optimum treatment outcomes. This work provides a question prompt list, co-produced with patients, that could be used as part of shared- decision making for initiation of anticoagulants in people with kidney disease, alongside a set of recommendations for prescribers.

## Supplementary Information

Supplementary Material 1.

Supplementary Material 2.

Supplementary Material 3.

## Data Availability

Anonymised patient quotes are available in the coding framework of supplementary appendix two, The data transcripts are not readily available to protect study participant privacy.
