# Supplementary material for: Facilitating active participation in anticoagulant decisions in advanced kidney disease: co-production of a question prompt list
Source: BMC Nephrol. 2025 Jan 28;26:42. doi: 10.1186/s12882-025-03966-y (PMC11773976; doi:10.1186/s12882-025-03966-y)
Supplement: Supplementary file 1 — Supplementary Material 1. [file 12882_2025_3966_MOESM1_ESM.docx]

Supplementary Appendix one. Focus group semi-structured topic guide

Topics for discussion

**At the time treatment was started**

1. Did you know why you needed to start blood thinners?
2. Were you made aware of the risks involved with blood thinners?
3. How well do you feel the risks and benefits of blood thinning treatment was explained to you?
4. Do you feel this information was presented to you in a way which made this decision easy or difficult to make – i.e. would you have liked it presented in writing or have had more time to think about it?
5. Did you feel you were involved with the decision to start blood thinners? Or did you prefer not to be?
6. Were you aware of the different options available and whether they would have been suitable for you?
7. How did you feel about starting anticoagulation?
8. From your experience what do you think would be useful for patients to know when they start blood thinners?
9. How do you think this should be presented?

**Ongoing treatment**

1. Does anyone regularly discuss your treatment with you to see how you are managing?
2. Were you given the option to review your decision of taking your blood thinners?

**Overall**

1. If you have had a good experience with the way your blood thinning drugs were managed what made it good?

If you have had a bad experience with your blood thinning drugs was there anything your kidney team could have done to make it better?
