# Supplementary material for: Facilitating active participation in anticoagulant decisions in advanced kidney disease: co-production of a question prompt list
Source: BMC Nephrol. 2025 Jan 28;26:42. doi: 10.1186/s12882-025-03966-y (PMC11773976; doi:10.1186/s12882-025-03966-y)
Supplement: Supplementary file 2 — Supplementary Material 2. [file 12882_2025_3966_MOESM2_ESM.docx]

| Theme | Sub-theme | Code | Quotes |
| --- | --- | --- | --- |
| Explanation of the problem | Indication | Understanding of the condition requiring treatment | That’s what they were saying, you know, if you don’t…if you don’t take these, um, you, you can get blood pooling in your heart that can cause a clot, and that clot can travel round your body and it can cause a stroke or, you know, a heart attack. |
|  |  |  | the first time I started, it was because I had a DVT in my right leg, so that was very clear. |
|  |  |  | I mean, when I was, when they found that I had a pulmonary embolism, they told me I was going on warfarin, to thin my blood, to avoid getting further blood clots, and to allow the clots that were there to break up. |
|  |  |  | Yeah, it was very clear, because I had a DVT. |
|  |  |  | the, the reason I’m taking warfarin is, is that I, I threw a PE whilst in hospital. |
|  | Duration | Understanding of length of treatment prescription | I had quite a, a lengthy conversation about the pros and cons. Because, inevitably, it was up to me whether I chose to stay on it. Um, so, so I think in my circumstances there was quite a good discussion, but it was so that I was fully informed in making that choice”. |
|  |  |  | It’s just an ongoing thing really, but no one ever discusses… You know, I assume it’s for life, but I don’t know, no one’s ever had that discussion really. |
|  |  |  | If they’re (patients) told it is going to be long-term, then, eh, then immediately push for, okay, this is going to have a large effect on my life. I want to be part of that journey. |
| Presentation of the options | Treatment options | Information on all treatment options | I think obviously, um, I just accepted that I needed to go on, on to it (warfarin), um, and I think no one ever… There was never any alternative discussed. |
|  |  |  | Well, you know, because basically, I, I was put on them but there was no other, um…no other discussions about alternatives at all, I was just told I need to go on warfarin. |
|  |  |  | I was just told, the only thing you can have on dialysis with your illness is warfarin. That’s it. |
|  |  |  | I think, somebody, another consultant said, why are you on warfarin, why aren’t you on this other drug, and when I said, oh you know, because of my kidney disease, and they said, oh yeah, of course. But I don't know why. |
|  |  |  | I didn’t feel, as a patient, that I was involved in the decision. And when I asked the question about an alternative, it was closed down. |
|  |  |  | I myself knew about apixaban, because myself, I needed to take apixaban for a while. But I asked why I couldn’t take that medication instead of warfarin, and they told me the reason was, because being a kidney patient, if there was any emergency, apixaban takes longer, and it's more difficult to stop the INR, or something like this. So, warfarin is easier, if they needed an intubation, or a surgery, or whatever, they can control it better, and turn it around easier, with warfarin. |
|  | Monitoring options | Burden to patient | I think the main thing I would have liked to have known was, eh, about the fact that I could do it myself. I found it an absolute pain the jacksie to, um, to keep going back and forth to the hospital before work, um, and it wasn’t necessary. |
|  |  |  | Um, my GP used to take my blood and then send it off to the hospital. And it, it was affecting me for work and everything because it was…I couldn’t…I, I couldn’t leave for work until I saw the GP. |
|  |  |  | Honestly, going back and forth to the doctor’s drives me mad. |
|  |  |  | I had to go to my local hospital, because they don't give you the option to do it in dialysis, and they don't give me the option, even to go to a GP which is closer to you. At least in my area, the only place I could do it, it was my local hospital, and that’s why I asked for the machine. Because to get to the hospital, I need to go on a bus, you know. And I was very tired, especially during COVID, and everything, being a high risk patient, I was freaking out every time I had to go in to my INR check. But there was nothing I could do, there were no options given to me, at all, whatsoever. |
|  |  |  | In my experience …. I lived on the other side of town to my hospital. And I didn’t drive. And of course, you have to get it done first thing in the morning, and I had to drop one child off at school, and bring the other one with me, and walk, through every weather that existed, for months, every single week, to go and get it done. Because my INR would not stabilise. And that’s probably, out of like all of the things that have happened to me, medically, that’s one of the hardest. Just simply accessing a blood test every week. |
|  |  | Availability of monitoring options for an individual | One thing about actually about who and where I can do this? I mean, you’re in and out like a yoyo on dialysis. I did dialysis in hospital and I was in there four times a week. Then I was going in doing bloods at that place because it had to be done there, apparently. And all the time, time wasted, and the, the amount of effort you had to make while in, being in the place you were on dialysis, was just crazy. Instead of…eventually somebody said, why don’t you just go to your GP and get a finger prick? Okay and that was the annoying thing, for so many years nobody said anything ”. |
|  |  |  | Yeah, the only, the only way that I found out that I could have it done it at dialysis was because I was in a situation where I was having surgery and they, they then said, we can do your INR. Because I was waiting for my INR to come down so they knew it was okay for surgery, so they, started doing it then, and then obviously I was aware of it. But before that, I wasn’t, I was going backwards and forwards to, to a clinic. … You know, it obviously makes it a lot easier … If they can just take a blood out of the dialysis line before you start, it’s not really a big deal. |
|  |  |  | …. there was times when I had to go every single week, for an INR check. And I would be so tired of it, so I requested to the doctor, if I could have the machine at home, and she said, yes of course, you can have the machine at home, it's no problem, you can just notify the results to the clinic. And when I told in the clinic that I wanted to have the machine, …. she said, but you need to have the training, and we don't have the time to train you, now. So they just completely block it out of the way, even if the doctor has said it's okay for you to have the machine at home, you know. So, all those options should be given to the patients when they start, and that they are aware that there is those possibilities, you know. |
|  |  | Financial cost of monitoring | Um, the test strips are available from the NHS as a standard prescription, but you do have to buy the machine… I bought a machine and it was four hundred pound thirty years ago. Um, but I’ve never bought once since because it’s always been replaced by a Roche. |
|  |  |  | the first thing that the nurse said, oh but the machine is every expensive. And I was like, okay. |
|  |  |  | Eh, the thing I don’t understand, given the return on investment, is why the NHS don’t buy them for people. I paid two hundred and ninety-nine pounds for mine. Um, I don’t know how many years ago now but quite a few. But I would really recommend it. |
| Discussion of risks and benefits | Factors affecting the risks of treatment | Female sex | But I did find, when I was going to the anticoagulation clinic regularly, and I would report bruising, or I'd show bruising, and I did actually have some internal bleeding. And they (nursing staff) didn’t seem particularly bothered about it. Because my INR was in range. |
|  |  |  | When I lost my kidney, I actually had my INR set in the clinic, and I said to the nurse, I'm going to start my period, my INR is too high, because my limit was 2.5, and it was already on 3. I said, my period is starting today, I'm going to bleed, and I'm really worried, and she said (the clinic nurse), oh don't worry, you should be fine because the INR is fine, it's not too bad. And then, by the time I managed to get to the clinic, my haemoglobin was 3.6 and it was too late. |
|  |  |  | Yeah, it's just such a familiar story.  Yeah, very upsetting when they don't (healthcare staff) listen to the patient, you know. |
|  |  |  | But you as a patient, really know what one thing does to the other, because you are, especially for women, our periods are very, very much affected by the warfarin. |
|  |  | Interactions | how it will affect my lifestyle (anticoagulant) and what I shouldn’t do that I did do a lot of, um, with alcohol. Nobody said anything until somebody said, you can’t drink that, you know that? Eh, no. |
|  |  |  | one time, the INR nurse said to me, oh you need to eat more vegetables, because you have anaemia ….I eat more greens, the next time I go for the check-up, my INR was under rate, because I had taken too much vegetables. But no one told me that the vitamin K, or whatever, could be like applied to too many vegetables, it could be affecting the INR, which I had no idea. And then she blamed me that, you cannot eat so many vegetables, and I was like, what? |
|  |  |  | So, probably, if there would have been any information at the time, I would have liked to be aware of the things like foods to avoid, and things like this, which I have learned through the years. |
|  |  |  | But, um, one of the things as well that I wasn’t told was that antibiotics affect INR. So I had a few experiences where my lines would clot on the machine and it was because I’d been on antibiotics for something else. So now, obviously now I know, I’m paranoid. If I go on antibiotics, I go every day to the doctor’s just to check, you know, how my INR is. And things like, knowing about, um, how other medications would affect it. |
|  |  | Bridging | Um, and I’m, I’m much more on it now. Obviously every surgery I have now I’m like, I’m on warfarin, when do I start my warfarin again? Do I need anything to, to bridge me? |
|  |  |  | Um, but then I also did my own bridging, so I also do all my own injections after surgery and everything as well, which again, they find a bit weird [laughs]. But, but I…that’s what I’m used to. |
|  |  |  | Eh, since starting dialysis, surgical procedures are a disaster. And as soon as I left one hospital my own anticoagulation group said, it’s nothing to do with us, we don’t do your bridging plans anymore. And the second hospital didn’t know anything about it. It ended up with a last minute bridging plan. Um, it’s still a problem. Every time I’ve had surgery… I had, um, cancer surgery, eh, last year and it ended up with about, um…I think the day before the operation, eh, me going in, speaking to the anaesthetist, and we prepared our own plan, our own bridging plan |
|  |  |  | I go to IntraHealth, which is an independent, I think, anticoagulation service, and they, they don’t… They say it’s not their responsibility to deal with bridging. And then you go to the hospital and then they say, it’s not really our…it’s not really our responsibility, ask the anticoagulation people. So they sort of ping you backwards and forwards between the two, saying… No one seems to want to take responsibility for that.  … It’s not going to happen to me again with…you know, with, with, um, that scenario of being sent out without bridging injections. I make sure that they, they do, do know and there’s, there’s a plan in place. |
| Professional recommendations |  | Communication of clinician’s view | I didn’t have a problem with understanding why I was being put on, eh, the blood thinners. I think what I found, though, was there was a very definite view from clinicians about, eh, what that looked like and the sort of service that you got offered. |
|  |  |  | It just wasn’t… In, in those days, it, it wasn’t a decision, it was just, this is what you’re taking. Um, so that was it. |
|  |  |  | Well, you know, because basically, I, I was put on them…. I accepted it at the time because of my health |
|  |  |  | I was basically told I had to go on it. I’d heard from other people, oh, you know, , I’m on warfarin. So I was a bit concerned about it, but I just basically accepted what the consultant said and I went on it. I didn’t question it at all but I, I didn’t particularly want to go any more tablets because obviously we’re all on so many anyway. |
| Check understanding or defer decision | Timing of information provision | Patient unable to fully engage | My first introduction. When I woke up after the operation was you’ve got a high, high risk factor of clotting, and if you do clot, then you’ve gone…you’re going to be in serious trouble with a lot of other complications. So that was that was pretty much it. |
|  |  |  | I just went in for a routine renal appointment and got taken into the emergency assessment unit and they said, you’ve got to go on warfarin. I was more concerned about getting to Malta than anything else I think. And as I say, on that day I was frantically [laughingly] just wanting to get on that plane and go to Malta [laughs]. And so probably I didn’t consider things as much as… |
|  |  |  | after I'd had the pacemaker inserted, I wasn’t so clear (on the reason for warfarin initiation). the reason why I had the pacemaker was because I was in AF. And I couldn’t quite get my head around everything, but it was at the end of a long period of hospitalisation, lots of things going on. |
|  | Follow up from Information provision | Support with decision making | if I read something, I panic. Instead of actually being able to sit with somebody and have somebody supporting me next to me, understanding it with me and being able to discuss it, that’s a better way. |
|  |  |  | I quite like some information to read and go away, digest it, and then come back and discuss. |
|  |  |  | I suspect the answer is, eh, that we need lots of different ways of supporting people. |
|  |  |  | Well obviously, my first one (clot) was when it was literally the birth of the internet, so nothing existed yet. So, it would have been great to have at least got a leaflet. |
| Arrange follow up | Initial follow-up | Patient preferences in relation to follow-up | I think discussions, an hour spent at the beginning would be beneficial, as I said, in my case going back. Um, I, I’ve come on this journey through discovery |
|  |  |  | And I feel like in hindsight, I think it would be great if, you know, that we were sent to an INR clinic, where you'd have an appointment, and you'd sit down with someone, they'd go through it all with you. They'd explain it all, and you'd have some literature to take away with you, as well as a website to look at. And talking through your options, and medication, and what it means. |
|  |  |  | All the information that you need to have, you learn it over time. But if you were taught in the beginning, like, okay this is your range, and this is where you should stay. |
|  | Long term follow-up | Ongoing patient support | I’m one of those people who googles everything and read everything I could anyway, but there might be other people who don’t. |
|  |  |  | if I read something… like google, if I read something, I panic. Instead of actually being able to sit with somebody and have somebody supporting me next to me, understanding it with me and being able to discuss it, that’s a better way. |
|  |  |  | I never had any appointments after my blood clot, I never had any follow-up clinics, or phone calls, or anything like that. I was just given the drug, and I was sent the appointment letter to go and have my bloods, and I had the little card to go in every week, and that was it. And that’s what I did for years. And I just put with all the symptoms. |
|  |  | Balance of risks and benefits over time | you know, how many bleeds does it take for somebody to actually do something….  You know, is now the time to look at an alternative, because the research has moved on. You know, being on warfarin permanently, what does that mean? It could be 20 years, a lot of things could happen in research in that period of time. So there should be an opportunity to review the anticoagulant, as well as how you're getting on. |
