# Supplementary material for: Facilitating active participation in anticoagulant decisions in advanced kidney disease: co-production of a question prompt list
Source: BMC Nephrol. 2025 Jan 28;26:42. doi: 10.1186/s12882-025-03966-y (PMC11773976; doi:10.1186/s12882-025-03966-y)
Supplement: Supplementary file 3 — Supplementary Material 3. [file 12882_2025_3966_MOESM3_ESM.docx]

Supplementary appendix three- Co-produced patient materials

**Anticoagulants for patients with kidney disease**

**Part 1- General Information**

**What are anticoagulants (“blood thinners”)?**

Anticoagulants work by affecting factors that your blood needs to clot, this means that your blood will take longer to form a blood clot.

Anticoagulants that are used in patients with kidney disease include warfarin, apixaban, rivaroxaban and edoxaban. The choice of anticoagulant depends on the reason you are taking an anticoagulant and how well your kidneys are functioning.

**Why might people with kidney disease need anticoagulants?**

People with kidney disease and a kidney transplant have an increased risk of developing blood clots. This may be related to specific kidney conditions but also other factors that can’t be fully explained.

People with kidney disease also have an increased chance of developing a fast irregular heart rate known as atrial fibrillation. This can occur in up to a quarter of patients on haemodialysis. Atrial fibrillation can lead to blood pooling in the heart and forming a clot, this clot can then break off and lead to a stroke.

Anticoagulants are most commonly used in the treatment of blood clots and to prevent stroke in patient with atrial fibrillation, but they can also be used in blood clot prevention.

Anticoagulants are different to antiplatelets such as aspirin or clopidogrel. Antiplatelets prevent blood cells known as platelets from clumping together and forming a clot, they are mainly taken by people who have had a heart attacks or stroke.

Your clinical team will explain the reason you are taking an anticoagulant and how long you will need to take it.

**What are the most common side effect of anticoagulants?**

The most common side effect of anticoagulants is that it takes you longer to stop bleeding, for example if you experience a cut then you may bleed for longer. Kidney disease may also contribute to increased bleeding.

If you experience a head injury you should seek urgent medical attention to make sure there is no bleeding in your brain.

Other types of serious bleeding you may experience which requires medical attention includes:

- Heavy bleeding during a period
- Bleeding in your stool or urine
- Coughing up blood
- Blood in your sick

**Part 2- Your personal anticoagulant regime**

This section allows you to fill in details and write notes relating to your own personal anticoagulant regime.

**My anticoagulant regime**

Drug:

Dose:

Reason for taking:

Duration:

Below are some examples of questions that you may wish to discuss with your clinical team when you are being started on anticoagulants. You can use this sheet to fill in the answers to the questions you ask during the discussion.

Date of conversation

Name of clinician

Why am I taking an anticoagulant?

How long will I need to take my anticoagulant for?

What kinds of anticoagulants can be prescribed for me?

What monitoring do I need to have, for example any specific blood tests?

Can I choose where this monitoring will be carried out?

What are the main side effects associated with my anticoagulant? When do I need to seek medical attention?

Does my diet or other medicines affect my anticoagulant?

What happens if I need a tooth removing or surgery?

Who will be responsible for following up on my treatment?

Who can I contact if I need help or advice? How do I contact them?

**Useful resources**

For further information about **warfarin** <https://www.medicines.org.uk/emc/rmm/1081/Document>

<https://patient.info/medicine/warfarin-an-anticoagulant>

<https://www.nhs.uk/medicines/warfarin/>

For further information about the direct oral acting-anticoagulants (apixaban, edoxaban, rivaroxaban)

**Apixaban** <https://patient.info/medicine/apixaban-tablets-eliquis>

<https://www.nhs.uk/medicines/apixaban/>

**Edoxaban** <https://patient.info/medicine/edoxaban-tablets-lixiana>

<https://www.nhs.uk/medicines/edoxaban/>

**Rivaroxaban** <https://patient.info/medicine/rivaroxaban-tablets-xarelto>

<https://www.nhs.uk/medicines/rivaroxaban/>
